# Metabolic health, menopause, and physical activity—a 4-year follow-up study

**DOI:** 10.1038/s41366-021-01022-x

**Published:** 2021-11-20

**Authors:** Matti Hyvärinen, Hanna-Kaarina Juppi, Sara Taskinen, Jari E. Karppinen, Sira Karvinen, Tuija H. Tammelin, Vuokko Kovanen, Pauliina Aukee, Urho M. Kujala, Timo Rantalainen, Sarianna Sipilä, Eija K. Laakkonen

**Affiliations:** 1 Gerontology Research Center and Faculty of Sport and Health Sciences, University of Jyväskylä, Jyväskylä, Finland; 2 Department of Mathematics and Statistics, University of Jyväskylä, Jyväskylä, Finland; 3 Faculty of Sport and Health Sciences, University of Jyväskylä, Jyväskylä, Finland; 4 LIKES Research Centre for Physical Activity and Health, Jyväskylä, Finland; 5 Department of Obstetrics and Gynecology, Pelvic Floor Research and Therapy Unit, Central Finland Health Care District, Jyväskylä, Finland

**Keywords:** Risk factors, Metabolic syndrome

## Abstract

**Background:**

In women, metabolic health deteriorates after menopause, and the role of physical activity (PA) in mitigating the change is not completely understood. This study investigates the changes in indicators of metabolic health around menopause and evaluates whether PA modulates these changes.

**Methods:**

Longitudinal data of 298 women aged 48–55 years at baseline participating in the ERMA and EsmiRs studies was used. Mean follow-up time was 3.8 (SD 0.1) years. Studied indicators of metabolic health were total and android fat mass, waist circumference, waist-to-hip ratio (WHR), systolic (SBP) and diastolic (DBP) blood pressure, blood glucose, triglycerides, serum total cholesterol, and high- (HDL-C) and low-density (LDL-C) lipoprotein cholesterol. PA was assessed by accelerometers and questionnaires. The participants were categorized into three menopausal groups: PRE-PRE (pre- or perimenopausal at both timepoints, *n* = 56), PRE-POST (pre- or perimenopausal at baseline, postmenopausal at follow-up, *n* = 149), and POST-POST (postmenopausal at both timepoints, *n* = 93). Analyses were carried out using linear and Poisson mixed-effect models.

**Results:**

At baseline, PA associated directly with HDL-C and inversely with LDL-C and all body adiposity variables. An increase was observed in total (*B* = 1.72, 95% CI [0.16, 3.28]) and android fat mass (0.26, [0.06, 0.46]), SBP (9.37, [3.34, 15.39]), and in all blood-based biomarkers in the PRE-POST group during the follow-up. The increase tended to be smaller in the PRE-PRE and POST-POST groups compared to the PRE-POST group, except for SBP. The change in PA associated inversely with the change in SBP (−2.40, [−4.34, −0.46]) and directly with the change in WHR (0.72, [0.05, 1.38]).

**Conclusions:**

In middle-aged women, menopause may accelerate the changes in multiple indicators of metabolic health. PA associates with healthier blood lipid profile and body composition in middle-aged women but does not seem to modulate the changes in most of the studied metabolic health indicators during the menopausal transition.

## Introduction

Metabolic health is an umbrella term for factors that combine several aspects of cellular, cardiovascular, and cardiorespiratory health and well-being. Body adiposity, anthropometrics, blood pressure, and blood-based biomarkers, such as serum lipids and blood glucose, can be clinically used to evaluate metabolic health. One established method is to use the diagnostic criteria of metabolic syndrome (MetS) [1], a multifaceted disorder predisposing individuals to severe health concerns, such as atherosclerotic heart disease [2] and type II diabetes [3]. Although there is a significant genetic component in the individual variance of metabolic health and emergence of MetS risk factors [4], unhealthy lifestyle habits, such as physical inactivity, are proposed to be a major contributor.

The effect of menopause on metabolic health and the development of MetS has been an increasing area of interest, as nowadays women in Western countries are expected to live in the postmenopausal state for more than one third of their lives [5–7]. Menopausal transition and the accompanying changes in the hormonal milieu (e.g., decrease in the systemic estradiol (E2) levels) have been associated with unfavorable changes in several indicators of metabolic health [8, 9]. For instance, increased blood glucose [10], accumulation of abdominal adiposity [11] as well as unhealthy changes in serum lipids [12] have been reported during menopausal transition. Additionally, menopause-related increase in inflammation marker levels [13] and decrease in muscle mass [14] have an additive negative impact on metabolic health. Therefore, it is not surprising that in women the incidence of MetS and cardiovascular disease increases after menopause [8, 15].

Physical activity (PA) has been widely proposed to improve the metabolic risk factor profile and cardiovascular health. Literature suggests that regular PA decreases total and visceral fat mass, improves insulin sensitivity, prevents dyslipidemia, and decreases systolic (SBP) and diastolic (DBP) blood pressure [16–18]. Thus, it can be used for the prevention and treatment of MetS. However, the associations between PA and changes in indicators of metabolic health around menopause are understudied as only few longitudinal studies have been conducted using device-measured PA [12, 19]. Moreover, these studies included only women transitioning from pre- or perimenopause to postmenopause and therefore could not address the contemporaneous aging-related changes.

The objective of this study was to investigate the changes around menopause in serum lipids and glucose, blood pressure, and body adiposity as indicators of metabolic health. Additionally, the aim was to evaluate whether PA modulates these changes using unique longitudinal data from the study of middle-aged women with different menopausal status.

## Materials and methods

### Study design and population

**Fig. 1:**
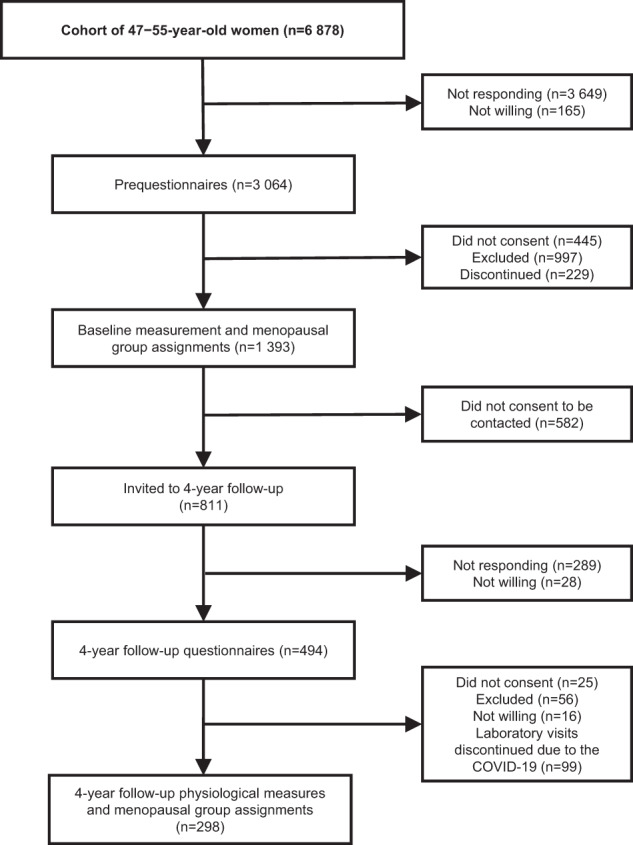
Flow chart of the study. The flow chart describes the participant enrollment and selection procedure of the ERMA and EsmiRs studies with detailed information about the exclusions and discontinuations during each phase of the study.

This study utilized the data from the observational Estrogenic Regulation of Muscle Apoptosis (ERMA) and Estrogen, MicroRNAs and the Risk of Metabolic Dysfunction (EsmiRs) studies. The participant selection for the ERMA study has been described in detail elsewhere [20]. Briefly, out of the 6 878 randomly selected women aged 47–55 years living in Central Finland, 1393 consented and met the inclusion criteria for the baseline measurements (Fig. 1). Exclusion criteria included conditions and the use of medications affecting ovarian function and systemic hormone or inflammatory status, such as bilateral oophorectomy, pregnancy, lactating, severe obesity (self-reported body mass index (BMI) ≥ 35 kg/m^2^), or the use of estrogen-containing medications and continuous cortisone or inflammatory drug treatment [20].

The 4-year follow-up measurements were carried out in the EsmiRs study. Out of the 811 participants measured in the ERMA baseline who consented to be contacted, 494 were willing to participate in the EsmiRs questionnaire. Of these participants, 56 were excluded, 25 did not consent, and 16 were not willing to continue to physiological measurements. The participants were excluded due to having more than 7 years from menopause based on the self-reports (*n* = 46), diabetes requiring regular insulin therapy (*n* = 2), severe cardiovascular dysfunction (*n* = 2), or diagnosed with cancer during the follow-up (*n* = 6). Furthermore, 99 participants could not be measured because of the COVID-19 lockdown. Consequently, the final study sample included 298 white women (Fig. 1). To estimate potential selection bias, sensitivity analyses comparing the included sample to the rest of the measured participants (*n* = 1095) at the ERMA baseline for all outcome variables and accelerometer-measured PA were conducted.

The recruiting for the ERMA study was conducted in 2014. The baseline measurements were initiated at the beginning of 2015, and they lasted until the end of 2016. The recruiting for the EsmiRs study started in November 2018 and laboratory measurements were initiated in January 2019. They were discontinued on March 16, 2020 due to the COVID-19 pandemic. The study was performed in accordance with the Declaration of Helsinki. All participants provided written informed consent, and the study was approved by the ethical committee of the Central Finland Health Care District (ERMA 8U/2014 and EsmiRs 9U/2018).

### Menopausal status assignments

Blood sampling after overnight fasting was performed in a supine position from the antecubital vein during days 1–5 of menstrual cycle if the cycle was predictable. Serum was separated from whole blood and stored at −80 °C before analysis. Serum concentrations of E2 and follicle-stimulating hormone (FSH) were determined using IMMULITE^®^ 2000 XPi (Siemens Healthineers, Erlangen, Germany) according to the manufacturer’s instructions.

Participants were categorized as pre-, peri-, or postmenopausal in both measurements based on the FSH concentrations and self-reported menstrual bleeding diaries using the adapted Stages of Reproductive Aging Workshop (STRAW + 10) guidelines [20]. The participants were divided into three groups based on how their menopausal status changed during the study. PRE-POST group (*n* = 149) consisted of women who experienced menopause during the follow-up period. That is, they were categorized as pre- or perimenopausal in the baseline and postmenopausal in the follow-up measurement. Furthermore, women that were pre- or perimenopausal (PRE-PRE, *n* = 56) or postmenopausal (POST-POST, *n* = 93) in both measurements were designated to their respective groups.

### Indicators of metabolic health

Blood pressure and anthropometrics were measured after overnight fasting. SBP and DBP was measured twice in a sitting position after 10 min rest using Omron M6 Comfort (Omron Healthcare, Kioto, Japan) with a standard size cuff and the mean values of the measurements were used. Waist circumference was measured in light underwear midway between the superior iliac spine and the lower rib margin, and hip circumference at the level of the greater trochanters [21]. Body mass and height were measured with standard procedures and BMI was computed by dividing the body mass with squared body height. Total body fat mass and percentage, android fat mass, and fat free mass were assessed with dual-energy X-ray absorptiometry (DXA; LUNAR, GE Healthcare, Chicago, IL, USA).

Serum samples collected during menopausal status assignment were also used for outcome variable analysis. Serum glucose, high- (HDL-C), low-density lipoprotein cholesterol (LDL-C), total cholesterol, and triglycerides were measured with KONELAB 20 XTi analyzer (Thermo Fischer Scientific, Vantaa, Finland).

The updated ATP III criteria for MetS risk factors was used [1]. The defining levels for risk factors were ≥88 cm for waist circumference, ≥130/≥85 mmHg for blood pressure, ≥1.69 mmol/l for serum triglycerides, ≥5.6 mmol/l for blood glucose, and <1.29 mmol/l for HDL-C.

### Physical activity

Accelerometry-measured PA was assessed in both timepoints by triaxial ActiGraph GT3X and wGT3X accelerometers (ActiGraph LLC, Pensacola, FL, USA) with an accompanied diary. Participants were instructed to wear the accelerometers for seven consecutive days on their right hip during waking hours, except during water-based activities. The data were collected at 60 Hz and the Euclidian norm of the resultant acceleration was computed for each timepoint. Consequently, the mean amplitude deviations (MAD) were computed for non-overlapping 5 s epochs, and the mean MAD value for 1 min epochs were determined based on the 5 s MAD values [22]. The accelerometer-measured MAD (ACC-MAD) reflects the directly measured acceleration and captures the volume of the activity in the entire intensity profile [23] and has been validated against oxygen consumption [24]. Non-wear time was identified as any epoch of at least 60 min with 1 min MAD continuously less than 0.001 g (g denotes the gravitational acceleration on Earth).^1^ A minimum of 3 days with a wear time of 10 h or more was regarded as a valid measurement.^2^ Finally, the ACC-MAD was determined for wear time for each measurement. For supplementary information, we defined activity with intensity higher or equal to 0.091 g as moderate-to-vigorous physical activity (MVPA) [24]. The ACC-MAD was strongly associated (*r* = 0.88 and *r* = 0.79) with the amount of MVPA and ActiGraph counts [25], respectively.

Additionally, PA was assessed by a self-reported questionnaire (SR-PA) [26]. Briefly, the questionnaire included four questions about the average frequency, intensity, and duration of leisure time PA bouts as well as the average duration of the commuting activity. Based on the responses, the metabolic equivalent (MET) hours per day for leisure time PA was calculated.

### Covariates

The use of medications and lifestyle habits were assessed by a structured questionnaire at baseline and follow-up measurements. Responses were used to assess alcohol consumption in portions per week and current smoking status (nonsmoker/smoker). Participants also reported their use of regular prescription medications that were categorized using the Anatomical Therapeutic Chemical (ATC) classification [27]. The use of medications was assessed (non-user/user) separately in preparations affecting blood pressure (ATC C02–05 and C07–09), serum lipids (ATC C10), and thyroid function (ATC H03).

Based on self-reports, participants were classified as being either non-user, only estrogen, only progestogen or combined estrogen and progestogen users. Exogenous sex hormone preparations for contraceptive and hormone replacement therapy use, such as pills, intra-uterine device, patches, and transdermal gels but not intravaginal local estrogen therapy were included. Diet quality score (DQS) was computed based on a food-frequency questionnaire as reported previously [14]. Shortly, DQS consisted of 11 elements characteristic to a healthy diet by the Nordic Nutrition Recommendations 2012. A higher intake of whole-grains, vegetables, fruits and berries, low-fat dairy, fish, and nuts and seeds, and a lower intake of processed grains, processed meats, sugary beverages, fast foods, and sweet or salty snacks were regarded as beneficial. Each component accounted for was worth of 1 point, and the maximum score available was therefore 11 points. A higher DQS score was regarded to reflect a healthier diet. The DQS was partly adapted from Masip et al. [28].

### Missing data

The percentage of missing values across the variables separately for each timepoint varied from 0 to 21%. The number of valid measurements for 298 participants in each variable is presented in Table 1. The total number of missing data values was 741 out of 13,708 (5%). Missing data occurred due to invalid or missing measurements as well as unclear or incomplete questionnaire responses. Missing data were assumed to occur at random and multiple imputation was used to create and analyse 50 multiply imputed data sets. Multiple imputation was carried out in R [29] using the “mice” package [30]. All variables measured at the same timepoint and the target variable measurement from the other timepoint were used for imputation of each variable. The number of iterations was set to 50 and passive imputation was used for the derived waist-to-hip ratio (WHR) variable. The model parameters were estimated in each imputed dataset separately and pooled using Rubin’s rules [31]. For comparison, we also performed the complete case analysis and there was no notable difference in the results that would have led to different conclusions.

**Table 1:** Characteristics of the study population in full sample and separately for each group.

	Full sample			PRE-POST			PRE-PRE			POST-POST		
	BL	FU	*Change*^*a*^	BL	FU	*Change*^*a*^	BL	FU	*Change*^*a*^	BL	FU	*Change*^*a*^
Age and blood-based biomarkers [*n*]	298	298	*298*	149	149	*149*	56	56	*56*	93	93	*93*
Age [year]	51.3 ± 1.8	55.1 ± 1.8	*3.8* ± *0.1*	51.3 ± 1.7	55.2 ± 1.7	*3.8* ± *0.2*	50.0 ± 1.4	53.8 ± 1.4	*3.8* ± *0.1*	52.1 ± 1.8	55.9 ± 1.8	*3.8* ± *0.1*
Estradiol [nmol/l]	0.38 ± 0.53	0.26 ± 0.28	*−0.12* ± *0.62*	0.47 ± 0.49	0.20 ± 0.21	*−0.27* ± *0.54*	0.52 ± 0.98	0.58 ± 0.39	*0.06* ± *1.09*	0.15 ± 0.10	0.17 ± 0.13	*0.02* ± *0.10*
Follicle-stimulating hormone [IU/l]	39.9 ± 37.1	69.5 ± 37.5	*29.5* ± *40.5*	24.2 ± 21.8	80.3 31.9	*56.0* ± *36.3*	11.7 ± 16.6	18.9 ± 12.3	*7.1* ± *21.5*	82.0 ± 29.1	82.6 ± 29.6	*0.6* ± *24.7*
Total cholesterol [mmol/l]	5.23 ± 0.91	5.67 ± 1.00	*0.43* ± *0.88*	5.14 ± 0.90	5.75 ± 1.02	*0.61* ± *0.75*	5.07 ± 0.78	5.41 ± 0.99	*0.34* ± *0.92*	5.50 ± 0.95	5.69 ± 0.94	*0.20* ± *0.98*
HDL-C [mmol/l]	1.72 ± 0.47	1.91 ± 0.50	*0.19* ± *0.39*	1.68 ± 0.42	1.93 ± 0.48	*0.25* ± *0.39*	1.61 ± 0.38	1.78 ± 0.41	*0.17* ± *0.29*	1.86 ± 0.55	1.97 ± 0.56	*0.11* ± *0.42*
LDL-C [mmol/l]	3.05 ± 0.80	3.41 ± 0.88	*0.37* ± *0.76*	2.98 ± 0.75	3.49 ± 0.91	*0.51* ± *0.67*	2.97 ± 0.75	3.27 ± 0.86	*0.30* ± *0.80*	3.20 ± 0.89	3.37 ± 0.85	*0.17* ± *0.82*
Glucose [mmol/l]	5.15 ± 0.45	5.16 ± 0.62	*0.02* ± *0.55*	5.12 ± 0.43	5.22 ± 0.70	*0.10* ± *0.64*	5.18 ± 0.42	5.20 ± 0.45	*0.03* ± *0.36*	5.18 ± 0.48	5.05 ± 0.55	*−0.13* ± *0.45*
Triglycerides [mmol/l]	1.08 ± 0.61	1.27 ± 0.73	*0.19* ± *0.53*	1.06 ± 0.53	1.31 ± 0.70	*0.25* ± *0.52*	1.03 ± 0.49	1.12±0.54	*0.10* ± *0.41*	1.13 ± 0.76	1.29 ± 0.88	*0.16* ± *0.60*
Blood pressure and anthropometrics [*n*]	249	298	*249*	139	149	*139*	46	56	*46*	64	93	*64*
Systolic blood pressure [mmHg]	132.0 ± 16.3	133.2 ± 18.3	*2.0* ± *13.4*	132.2 ± 17.4	133.6 ± 18.0	*2.0* ± *13.4*	132.0 ± 15.8	133.6 ± 19.1	*2.2* ± *13.6*	131.4 ± 14.6	132.2 ± 18.4	*1.8* ± *13.4*
Diastolic blood pressure [mmHg]	84.1 ± 9.2	81.9 ± 10.0	*−2.1* ± *6.5*	83.9 ± 9.7	82.2 ± 10.4	*−1.5* ± *7.1*	84.3 ± 8.6	81.5 ± 9.3	*−3.1* ± *5.9*	84.3 ± 8.8	81.7 ± 10.0	*−2.8* ± *5.3*
Waist circumference [cm]	82.9 ± 9.7	83.7 ± 10.4	*1.2* ± *4.2*	83.0 ± 10.3	83.8 ± 11.1	*1.3* ± *3.9*	83.2 ± 8.8	84.6 ± 9.6	*0.9* ± *4.8*	82.3 ± 9.0	83.1 ± 9.8	*1.1* ± *4.4*
Waist-to-hip ratio × 100	82.5 ± 6.4	84.2 ± 5.5	*1.7* ± *3.7*	82.4 ± 6.7	83.8 ± 5.4	*1.4* ± *4.1*	82.8 ± 6.0	85.1 ± 5.8	*2.1* ± *3.7*	82.7 ± 6.0	84.4 ± 5.4	*2.2* ± *2.5*
Weight [kg]	69.5 ± 10.8	70.9 ± 11.5	1.8 ± 3.9	69.8 ± 11.0	71.7 ± 12.3	2.3 ± 3.6	70.0 ± 10.3	72.0 ± 10.4	1.5 ± 3.3	68.3 ± 10.8	69.1 ± 10.6	1.0 ± 4.5
Body mass index [kg/m^2^]	25.3 ± 3.7	25.8 ± 4.1	0.7 ± 1.4	25.5 ± 3.9	26.2 ± 4.4	0.9 ± 1.4	25.1 ± 3.1	25.9 ± 3.3	0.5 ± 1.2	24.9 ± 3.6	25.3 ± 3.8	0.4 ± 1.6
Body mass index [kg/m^2^]^b^												
<18.5	0 (1)	1 (3)		0 (0)	1 (1)		0 (0)	0 (0)		2 (1)	2 (2)	
18.5–24.9	53 (131)	44 (131)		51 (71)	44 (66)		52 (24)	38 (21)		56 (36)	47 (44)	
25–29.9	36 (90)	39 (117)		36 (50)	36 (54)		39 (18)	48 (27)		34 (22)	39 (36)	
≥30	11 (27)	16 (47)		13 (18	19 (28)		9 (4)	14 (8)		8 (5)	12 (11)	
Body composition [*n*]	244	292	*240*	137	145	*134*	44	55	*44*	63	92	*62*
Total fat mass [kg]	24.2 ± 8.4	25.9 ± 9.1	*2.0* ± *3.3*	24.7 ± 8.9	26.7 ± 9.8	*2.6* ± *2.8*	23.5 ± 7.3	25.1 ± 7.9	*1.2* ± *3.3*	23.7 ± 7.9	25.1 ± 8.4	*1.3* ± *4.0*
Android fat mass [kg]	2.14 ± 0.91	2.39 ± 1.01	*0.27* ± *0.42*	2.18 ± 0.96	2.47 ± 1.09	*0.35* ± *0.38*	2.06 ± 0.81	2.26 ± 0.87	*0.15* ± *0.35*	2.11 ± 0.88	2.34 ± 0.96	*0.17* ± *0.50*
Total fat percentage [%]	34.0 ± 7.4	35.7 ± 7.6	2.0 ± 2.8	34.3 ± 8.0	36.4 ± 8.0	2.5 ± 2.3	32.9 ± 5.9	34.2 ± 6.6	1.1 ± 3.0	34.1 ± 7.0	35.5 ± 7.4	1.4 ± 3.3
Fat free mass [kg]	45.2 ± 4.3	44.8 ± 4.4	−0.4 ± 1.5	45.2 ± 4.3	44.6 ± 4.5	−0.5 ± 1.6	46.6 ± 4.5	46.8 ± 4.0	0.1 ± 1.3	44.2 ± 4.0	44.0 ± 4.2	−0.5 ± 1.5
Metabolic syndrome risk factors^b^ [*n*]	249	298		139	149		46	56		64	93	
0	31 (77)	29 (87)		31 (43)	28 (42)		28 (13)	29 (16)		33 (21)	31 (29)	
1	32 (79)	35 (103)		30 (42)	35 (52)		37 (17)	39 (22)		31 (20)	31 (29)	
2	22 (54)	21 (61)		24 (33)	20 (29)		15 (7)	18 (10)		22 (14)	24 (22)	
3	10 (25)	8 (25)		10 (14)	8 (12)		9 (4)	11 (6)		11 (7)	8 (7)	
4	4 (11)	5 (15)		4 (6)	6 (9)		7 (3)	4 (2)		3 (2)	4 (4)	
5	1 (3)	2 (7)		1 (1)	3 (5)		4 (2)	0 (0)		0 (0)	2 (2)	
Accelerometer-measured PA [*n*]	235	283	*222*	134	141	*126*	43	55	*43*	58	87	*53*
ACC-MAD [mg]	30.2 ± 10.0	28.3±8.6	*−1.9* ± *7.2*	29.7 ± 11.1	28.0 ± 8.3	*−1.8* ± *7.8*	31.7 ± 7.8	29.1 ± 8.8	*−3.3* ± *5.8*	30.3 ± 8.6	28.4 ± 9.0	*−0.9* ± *6.7*
Use of hormonal preparations^b^ [*n*]	298	298		149	149		56	56		93	93	
Non-user	62 (186)	60 (180)		62 (101)	66 (99)		39 (22)	34 (19)		68 (63)	67 (62)	
Progestogen	38 (112)	19 (56)		32 (48)	15 (23)		61 (34)	41 (23)		32 (30)	11 (10)	
Estrogen	0 (0)	3 (10)		0 (0)	3 (4)		0 (0)	4 (2)		0 (0)	4 (4)	
Progestogen + Estrogen	0 (0)	18 (52)		0 (0)	15 (23)		0 (0)	21 (12)		0 (0)	18 (17)	
Lifestyle habits [*n*]	276	298	*276*	144	149	*144*	53	56	*53*	79	93	*79*
Alcohol consumption [portions/wk]	3.73 ± 3.92	3.24 ± 3.43	*−0.53* ± *2.63*	3.93 ± 3.32	3.68 ± 3.69	*−0.26* ± *2.32*	3.00 ± 2.43	2.54 ± 1.94	*−0.58* ± *1.91*	3.86 ± 5.43	2.98 ± 3.62	*−0.99* ± *3.45*
Diet quality score	5.87 ± 2.45	5.85 ± 2.26	*−0.02* ± *1.90*	5.84 ± 2.52	5.70 ± 2.33	*−0.04* ± *1.90*	5.77 ± 2.28	6.20 ± 2.34	*0.38* ± *1.91*	5.99 ± 2.46	5.88 ± 2.08	*−0.27* ± *1.89*
Smoking^b^												
Non-smoker	95 (262)	94 (280)		95 (136)	94 (140)		96 (51)	96 (54)		95 (75)	92 (86)	95 (262)
Smoker	5 (13)	6 (18)		5 (7)	6 (9)		4 (2)	4 (2)		5 (4)	8 (7)	5 (13)

Data are mean ± SD unless otherwise specified.

*PRE-POST* participants who were pre- or perimenopausal at baseline and postmenopausal at follow-up, *PRE-PRE* participants who were pre- or perimenopausal in both measurements, *POST-POST* participants who were postmenopausal already at baseline, *BL* baseline measurement, *FU* follow-up measurement, *HDL-C* high-density lipoprotein cholesterol, *LDL-C* low-density lipoprotein cholesterol, *PA* physical activity, *ACC-MAD* accelerometer-measured mean amplitude deviation, *mg* milligravity (0.00981 m/s^2^).

^a^For participants with baseline and follow-up measurement.

^b^Data are % (*n*).

### Statistical analysis

The main analyses were carried out using linear and Poisson mixed-effect models with random intercept [32]. For each outcome variable, the fixed effects were time (0 = baseline, 1 = follow-up), menopausal group, ACC-MAD, and interactions between time and group as well as time and ACC-MAD. The interactions were included in the models to study how the change in PA associate with the change in outcome variables during the follow-up. Furthermore, the covariates included as fixed effects were mean centered age at baseline and the use of hormonal preparations. Residual plots, Q–Q plots, and correlation analysis were used for testing the model assumptions. The analyses were carried out in R using the “nlme” [33] and “lme4” [34] packages.

Based on the literature, we identified candidate covariates related to lifestyle habits and the use of medications that may be associated with the outcome variables. Their distributions in the study population are presented in detail in Supplementary Table 1. However, to our consideration, lifestyle habits and the use of antihypertensives, lipid-modifying agents or thyroid therapy do not significantly affect the progression of menopausal transition, and the use may even be caused by the menopause-induced changes in the outcome variables. Thus, only the use of sex hormone therapy was controlled for confounding. Nonetheless, we also performed the analysis including the relevant variables and their interaction with time as covariates, but it did not have a notable effect on the results. Furthermore, we conducted sensitivity analyses for blood lipids and blood pressure by excluding the participants who used lipid-modifying agents and antihypertensives, respectively.

## Results

### Characteristics of the study population

The average follow-up-time was 3.8 years in all groups (Table 1). At baseline, the participants were slightly overweight with mean BMI of 25.3 ± SD 3.7 and had slightly elevated SBP (132.0 ± 3.7), DBP (84.1 ± 9.2), total cholesterol (5.23 ± 0.91), and LDL-C (3.05 ± 0.80). Other outcome variable means were within the normal range [35, 36]. Participants in the PRE-PRE group were the youngest and had the lowest FSH and highest E2 levels at baseline. Respectively, the participants in the POST-POST group were the oldest and had the highest FSH and lowest E2 levels. The most notable changes in E2 and FSH levels occurred in the PRE-POST group during the follow-up. The percentage of the participants with three or more MetS risk factors was 16% at baseline and at follow-up. The sensitivity analyses using unpaired *T*-test indicated the study sample to have slightly lower blood glucose (5.15 ± 0.45 and 5.28 ± 0.63, *t* (1387) = 3.319, *p* = 0.001) and higher ACC-MAD (30.2 ± 10.0 and 28.8 ± 8.8, *t* (782) = −2.044, *p* = 0.041) compared to participants that did not participate in the follow-up. No differences were observed for other outcome variables (data not shown).

### Blood-based biomarkers

The PRE-POST group had lower total cholesterol and HDL-C compared to the POST-POST group (Table 2). In the full sample, ACC-MAD was directly associated with HDL-C (*B* = 0.06, 95% CI [0.01, 0.11]) and inversely with LDL-C (*B* = −0.11, 95% CI [−0.21, −0.01]). The levels of all blood-based biomarkers increased during the follow-up in the PRE-POST group and the increase tended to be smaller in the PRE-PRE and, especially, in the POST-POST group. The change in ACC-MAD was not associated with the change in any of the outcome variables measured from blood. The use of progestogen was associated with lower HDL-C, while the combined progestogen and estrogen use was associated with a lower blood glucose. The results did not differ notably when using SR-PA as a PA measure (Supplementary Table 2) or excluding participants using lipid-modifying agents (Supplementary Table 3).

**Table 2:** Pooled fixed effect estimates for blood-based biomarkers (*n* = 298).

	Total cholesterol [mmol/l]		HDL-C [mmol/l]		LDL-C [mmol/l]		Glucose [mmol/l]		Triglycerides [mmol/l]	
	*B*	95% CI	*B*	95% CI	*B*	95% CI	*B*	95% CI	*B*	95% CI
Intercept (PRE-POST)	5.48***	[5.12, 5.83]	1.54***	[1.38, 1.72]	3.33***	[3.02, 3.62]	5.17***	[4.97, 5.38]	1.24***	[0.99, 1.48]
*Main effects*										
Group										
PRE-POST (ref.)	–		–		–		–		–	
PRE-PRE	0.03	[−0.27, 0.33]	−0.05	[−0.20, 0.10]	0.05	[−0.22, 0.32]	0.04	[−0.13, 0.22]	0.02	[−0.19, 0.24]
POST-POST	0.33*	[0.09, 0.59]	0.18**	[0.05, 0.31]	0.21	[−0.02, 0.43]	0.06	[−0.08, 0.20]	0.05	[−0.13, 0.22]
ACC-MAD [10 mg]	−0.10	[−0.21, 0,01]	0.06*	[0.01, 0.11]	−0.11*	[−0.21, −0.01]	−0.02	[−0.08, 0.04]	−0.06	[−0.13, 0.01]
Age at baseline [year]	0.03	[−0.03, 0.09]	−0.00	[−0.03, 0.03]	0.02	[−0.03, 0.07]	−0.00	[−0.03, 0.03]	0.03	[−0.01, 0.07]
Use of hormonal preparations										
Non-user (ref.)	–		–		–		–		–	
Progestogen	−0.14	[−0.32, 0.04]	−0.11**	[−0.20, −0.03]	−0.05	[−0.21, 0.10]	0.06	[−0.05, 0.16]	−0.03	[−0.15, 0.09]
Estrogen	−0.19	[−0.68, 0.30]	0.16	[−0.07, 0.38]	−0.16	[−0.59, 0.27]	0.12	[−0.18, 0.42]	−0.13	[−0.45, 0.18]
Progestogen + Estrogen	−0.17	[−0.42, 0.07]	−0.08	[−0.19, 0.03]	−0.15	[−0.37, 0.06]	−0.19*	[−0.33, −0.04]	−0.02	[−0.17, 0.14]
Time (PRE-POST)	0.45*	[0.07, 0.84]	0.35***	[0.18, 0.52]	0.40*	[0.06, 0.74]	0.32**	[0.08, 0.55]	0.28*	[0.03, 0.52]
*Interactions*										
Time × Group										
Time × PRE-POST (ref.)	–		–		–		–		–	
Time × PRE-PRE	−0.28*	[−0.54, −0.01]	−0.07	[−0.18, 0.05]	−0.21	[−0.44, 0.02]	−0.06	[−0.22, 0.11]	−0.15	[−0.32, 0.01]
Time × POST-POST	−0.42***	[−0.65, −0.20]	−0.15*	[−0.24, −0.05]	−0.34**	[−0.53, −0.14]	−0.22*	[−0.36, −0.08]	−0.09	[−0.23, 0.05]
Time × ACC-MAD	0.05	[−0.07, 0.18]	−0.04	[−0.09, 0.02]	0.04	[−0.07, 0.15]	−0.07	[−0.14, 0.01]	−0.01	[−0.09, 0.07]

*HDL-C* high-density lipoprotein cholesterol, *LDL-C* low-density lipoprotein cholesterol, *CI* Confidence interval, *PRE-POST* participants who were pre- or perimenopausal at baseline and postmenopausal at follow-up (reference group), *PRE-PRE* participants who were pre- or perimenopausal in both measurements, *POST-POST* participants who were postmenopausal already at baseline, *ACC-MAD* accelerometer-measured mean amplitude deviation, *mg* milligravity (0.00981 m/s^2^), *Time* from baseline to follow-up.

*p ≤ 0.05; **p ≤ 0.01; ***p < 0.001.

### Body composition and anthropometrics

ACC-MAD was inversely associated with total fat mass (*B* = −0.77, 95% CI [−1.27, −0.26]) and android fat mass (*B* = −0.12, 95% CI [−0.18, −0.03]), waist circumference (*B* = −0.92, 95% CI [−1.60, −0.24]), and WHR (*B* = −0.89, 95% CI [−1.51, −0.27]) in the full sample (Table 3). Total (*B* = 1.72, 95% CI [0.16, 3.28]) and android fat mass (*B* = 0.26, 95% CI [0.06, 0.46]) increased during the follow-up in the PRE-POST group and the change was smaller in the PRE-PRE and POST-POST groups compared to the PRE-POST group. The change in ACC-MAD was directly associated with the change in WHR (*B* = 0.72, 95% CI [0.05, 1.38]). Combined progestogen and estrogen use was associated with lower android fat mass when compared to non-hormone users. The results were relatively similar when using SR-PA, however, SR-PA was not associated with the change in WHR (Supplementary Table 4).

**Table 3:** Pooled fixed effect estimates for body composition and anthropometrics (*n* = 298).

	Fat mass [kg]		Android fat mass [kg]		Waist circumference [cm]		Waist-to-hip ratio × 100	
	*B*	95% CI	*B*	95% CI	*B*	95% CI	*B*	95% CI
Intercept (PRE-POST)	26.66***	[24.59, 28.73]	2.46***	[2.19, 2.73]	85.41***	[82.81, 88.00]	84.95***	[82.85, 87.04]
*Main effects*								
Group								
PRE-POST (ref.)	–		–		–		–	
PRE-PRE	0.46	[−2.33, 3.26]	0.04	[−0.27, 0.35]	1.96	[−1.30, 5.22]	1.10	[−0.92, 3.12]
POST-POST	−0.74	[−3.05, 1.57]	−0.04	[−0.30, 0.21]	−0.75	[−3.48, 1.97]	−0.05	[−1.78, 1.68]
ACC-MAD [10 mg]	−0.77**	[−1.27, −0.26]	−0.11**	[−0.18, −0.03]	−0.92**	[−1.60, −0.24]	−0.89**	[−1.51, −0.27]
Age at baseline [year]	0.40	[−0.18, 0.98]	0.04	[−0.02, 0.11]	0.61	[−0.07, 1.29]	0.32	[−0.08, 0.72]
Use of hormonal preparations								
Non-user (ref.)	–		–		–		–	
Progestogen	−0.61	[−1.55, 0.34]	−0.06	[−0.18, 0.05]	−0.28	[−1.48, 0.92]	−0.12	[−1.19, 0.94]
Estrogen	1.02	[−1.08, 3.13]	0.09	[−0.17, 0.36]	1.14	[−1.58, 3.86]	0.19	[−2.37, 2.75]
Progestogen + Estrogen	−0.60	[−1.81, 0.62]	−0.14	[−0.29, 0.01]	−0.47	[−1.94, 0.99]	0.58	[−0.76, 1.92]
Time (PRE-POST)	1.72*	[0.16, 3.28]	0.26**	[0.06, 0.46]	0.44	[−1.64, 2.51]	−0.75	[−2.77, 1.26]
*Interactions*								
Time × Group								
Time × PRE-POST (ref.)	–		–		–		–	
Time × PRE-PRE	−1.33*	[−2.41, −0.26]	−0.15*	[−0.29, −0.02]	−0.19	[−1.61, 1.22]	0.61	[−0.81, 2.03]
Time × POST-POST	−1.20*	[−2.12, −0.28]	−0.12	[−0.24, 0.00]	−0.47	[−1.76, 0.81]	0.36	[−0.92, 1.64]
Time × ACC-MAD	0.24	[−0.28, 0.75]	0.03	[−0.04, 0.09]	0.23	[−0.46, 0.91]	0.72*	[0.05, 1.38]

*CI* Confidence interval, *PRE-POST* participants who were pre- or perimenopausal at baseline and postmenopausal at follow-up (reference group), *PRE-PRE* participants who were pre- or perimenopausal in both measurements, *POST-POST* participants who were postmenopausal already at baseline, *ACC-MAD* accelerometer-measured mean amplitude deviation, *mg* milligravity (0.00981 m/s^2^), *Time* from baseline to follow-up.

*p ≤ 0.05; **p ≤ 0.01; ***p < 0.001.

### Blood pressure

ACC-MAD was not associated with SBP and DBP in the full sample (Table 4). SBP increased during the follow-up in the PRE-POST group (*B* = 9.37, 95% CI [3.34, 15.39]) and the change did not differ between the groups. Additionally, the change in ACC-MAD was inversely associated with the change in SBP (*B* = −2.40, 95% CI [−4.34, −0.46]), but this association was not observed with SR-PA (Supplementary Table 5). The combined progestogen and estrogen use was associated with lower SBP and DBP. The results did not differ notably when excluding participants using antihypertensives (Supplementary Table 6).

**Table 4:** Pooled fixed effect estimates for blood pressure (*n* = 298).

	Systolic blood pressure [mmHg]		Diastolic blood pressure [mmHg]	
	*B*	95% CI	*B*	95% CI
Intercept (PRE-POST)	130.91***	[125.01, 136.80]	84.90***	[81.83, 87.96]
*Main effects*				
Group				
PRE-POST (ref.)	–		–	
PRE-PRE	1.31	[−4.41, 7.03]	1.10	[−2.07, 4.27]
POST-POST	−1.97	[−6.79, 2.85]	0.01	[−2.66, 2.68]
ACC-MAD [10 mg]	0.28	[−1.42, 2.00]	−0.36	[−1.24, 0.51]
Age at baseline [year]	1.10	[−0.03, 2.23]	0.42	[−0.22, 1.06]
Use of hormonal preparations				
Non-user (ref.)	–		–	
Progestogen	0.46	[−2.66, 3.59]	−0.18	[−1.80, 1.43]
Estrogen	1.76	[−6.24, 9.76]	−0.95	[−4.91, 3.01]
Progestogen + Estrogen	−5.55**	[−9.61, −1.49]	−4.33***	[−6.36, −2.30]
Time (PRE-POST)	9.37**	[3.34, 15.39]	−0.16	[−3.11, 2.79]
*Interactions*				
Time × Group				
Time × PRE-POST (ref.)	–		–	
Time × PRE-PRE	0.62	[−3.67, 4.91]	−0.81	[−2.88, 1.26]
Time × POST-POST	−0.03	[−3.80, 3.73]	−0.73	[−2.57, 1.12]
Time × ACC-MAD	−2.40*	[−4.34, −0.46]	−0.28	[−1.24, 0.68]

*CI* Confidence interval, *PRE-POST* participants who were pre- or perimenopausal at baseline and postmenopausal at follow-up (reference group), *PRE-PRE* participants who were pre- or perimenopausal in both measurements, *POST-POST* participants who were postmenopausal already at baseline, *ACC-MAD* accelerometer-measured mean amplitude deviation, *mg* milligravity (0.00981 m/s^2^), *Time* from baseline to follow-up.

*p ≤ 0.05; **p ≤ 0.01, ***p < 0.001.

### Number of MetS risk factors

In the Poisson mixed-effect models (Table 5), age at baseline was directly associated with the number of MetS risk factors at baseline (exp(B) = 1.07, 95% CI [1.00, 1.14]) in the full sample. The number of risk factors at baseline and the change in the number of risk factors during the follow-up did not differ between the groups. Furthermore, ACC-MAD was not associated with the number of risk factors at baseline nor with the change in the number. The results did not differ notably when using SR-PA (Supplementary Table 7) or excluding participants using lipid-modifying agents or antihypertensives (Supplementary Table 8).

**Table 5:** Pooled fixed effect estimates for number of metabolic syndrome risk factors (*n* = 298).

	Number of metabolic syndrome risk factors	
	exp (B)	95% CI
Intercept (PRE-POST)	1.41	[0.91, 2.16]
*Main effects*		
Group		
PRE-POST (ref.)	–	
PRE-PRE	1.31	[0.93, 1.84]
POST-POST	0.95	[0.71, 1.28]
ACC-MAD [10 mg]	0.91	[0.79, 1.04]
Age at baseline [year]	1.07*	[1.00, 1.14]
Use of hormonal preparations		
Non-user (ref.)	–	
Progestogen	0.98	[0.78, 1.23]
Estrogen	0.85	[0.41, 1.74]
Progestogen + Estrogen	0.71	[0.50, 1.02]
Time (PRE-POST)	1.59	[0.93, 2.73]
*Interactions*		
Time × Group		
Time × PRE-POST (ref.)	–	
Time × PRE-PRE	0.77	[0.53, 1.14]
Time × POST-POST	0.93	[0.67, 1.30]
Time × ACC-MAD	0.88	[0.73, 1.06]

*CI* Confidence interval, *PRE-POST* participants who were pre- or perimenopausal at baseline and postmenopausal at follow-up (reference group), *PRE-PRE* participants who were pre- or perimenopausal in both measurements, *POST-POST* participants who were postmenopausal already at baseline, *ACC-MAD* accelerometer-measured mean amplitude deviation, *mg* milligravity (0.00981 m/s^2^), *Time* from baseline to follow-up.

*p ≤ 0.05; **p ≤ 0.01; ***p < 0.001.

## Discussion

In this longitudinal study of middle-aged women, an increase in several indicators of metabolic health, ranging from blood-based biomarkers and SBP to body adiposity, were observed during the follow-up. The increase was greater during menopausal transition, and the rate of change decelerated after menopause, especially in blood-based biomarkers. Higher PA was associated with favorable levels in metabolic health indicators; however, the change in PA did not associate with the rate of change during the follow-up in most of the studied metabolic health indicators. Nonetheless, associations of higher PA with a greater increase in WHR and a smaller increase in SBP were observed. PA was not associated with the number of MetS risk factors.

We observed a significant increase in total cholesterol, HDL-C, LDL-C, triglycerides, and blood glucose in women going through menopause during the follow-up. Several other longitudinal studies have also reported an increase in serum total cholesterol, LDL-C, and triglycerides during the menopausal transition [37–40]. However, the literature on the associations of menopause and HDL-C is more inconsistent. Previous studies have reported HDL-C to increase [12, 19, 39, 41], peak right before menopause [40], as well as continuously decline during menopausal transition [42]. In addition to increase in HDL-C in the PRE-POST group, higher baseline HDL-C levels and lower increase rate in the postmenopausal group were also observed. These conflicting results suggest that the change in HDL-C during menopausal transition is a complicated process related to, e.g., aging and genetic background. As HDL-C and its antiatherogenic functionality have a major role in promoting cardiovascular health, it is obvious that more detailed longitudinal studies are needed to clarify this process.

Previous findings on associations of menopausal transition and blood glucose are also contradictory. Some longitudinal studies have reported a decrease [15, 19] during the menopausal transition, but in cross-sectional design postmenopausal women have been reported to have higher blood glucose compared to pre- and perimenopausal women [10, 43]. We observed an increase in fasting blood glucose in women going through menopause and the increase was attenuated in the POST-POST group. Our findings indicate that in addition to aging, the increase in blood glucose may be explained by the decreasing E2 levels during menopausal transition, since E2 is known to enhance insulin sensitivity and glucose disposal in women [44].

The observed increase in total and android fat masses in this study are consistent with previous literature [45–47]. The decrease in female sex hormone levels during menopausal transition is proposed to lead to increased accumulation of adipose tissue especially in the waist and visceral area [11, 48], yet the association of menopause to total adipose tissue accumulation is somewhat debated [9]. Although android fat mass increased during the follow-up, we did not observe a change in waist circumference. Similar results have also been reported by others [19, 49]. This indicates a change in the ratio between android lean and fat masses during the follow-up. A comparable change in muscle-to-fat ratio is also observed in total body level during the menopausal transition [11, 47]. Furthermore, we observed an increase in SBP that did not differ between the groups. This finding is supported by the previous review by Taddei [50] that suggested the changes in SBP to be more dependent on age than menopausal status in middle-aged women.

Regular PA is a well-established contributor to a healthier blood lipid profile and body composition also in menopausal women [12, 51]. With both accelerometry-measured and self-reported measures, higher PA was associated with lower levels in blood-based biomarkers and body composition variables but, surprisingly [52], not in blood pressure. When exploring the combined effect of PA and follow-up time, increased PA was associated with an accelerated increase in WHR. This result suggests accelerated decrease in hip circumference in more active women, since the change in PA was not associated with the change in waist circumference. While estradiol levels are associated with both gluteal adipose [53] and muscle mass [14, 54], we suspect that the pronounced decrease in more physically active women is caused especially by the loss of muscle mass due to the potentially higher muscle mass on their gluteal area at baseline. However, in the current study, we were not able to accurately identify the lost tissue type at the hip area. We also observed higher PA to be associated with a smaller increase in SBP during the follow-up. As discussed earlier, the observed changes in SBP may have been related to aging rather than menopausal transition [50], but our results indicate that regular PA may be efficient for controlling SBP in menopausal women similar to other populations [55, 56].

Although PA was associated with individual indicators of metabolic health, no associations with PA and the number of MetS risk factors or change in the number were observed. This may be caused by the strict cutoff points used in the clinical identification of MetS that does not capture the change unless the cutoff point is reached. Our findings are somewhat contradictory to a recent longitudinal study [57] in which higher PA was associated with lower incidence and better recovery from MetS in middle-aged women. Nonetheless, also in our study, the number of MetS factors tended to be smaller and the increase in the number was slightly lower in more active participants. Thus, PA might be beneficial for preventing the unwanted changes in individual MetS risk factors, but more studies on the associations of PA and number of MetS risk factors during menopause are required.

An interesting additional finding of the study was the observed associations of external hormone use with multiple indicators of metabolic health, highlighted by the distinctive association between the combined use of estrogen and progestogen and lower SBP. The use of hormone replacement therapy has been previously shown to reduce abdominal fat, blood glucose, LDL-to-HDL ratio and blood pressure [58], similar to our results. The individual effects of progestogen use on body composition and metabolic health are less studied, but estrogen is recognized to associate directly with gynoid adipose tissue volume [48, 59], better insulin sensitivity [44], and beneficial effects on vasodilatation and LDL-C concentration [60]. Although our results from exogenous hormone use are mostly in agreement with previous results, the results need to be interpreted with caution, since we did not consider the dosage, the duration of use, or form of the exogenous hormones.

One of the limitations was that the measurements were repeated only once. The homogenous sample of white, middle-aged women with exclusion of women with severe obesity and different medical disorders may limit the generalizability of the results for more heterogeneous populations including participants with disabling conditions. Furthermore, based on the sensitivity analysis, dropouts during the study have caused healthy selection bias particularly towards slightly better glucose control and higher PA which also limits the generalizability of the results. However, this is unlikely to have caused overestimation of the observed unhealthy menopause-related changes in outcome variables. The strengths of the study included the use of accelerometers for PA and DXA for body composition measurements. Additionally, the study design in which women of similar age but different menopausal status were followed for the same amount of time allowed to study the menopause-related changes in outcome variables while taking into account the simultaneous aging.

In conclusion, the results indicate that undesirable changes in blood lipids, body adiposity, and blood pressure occur in middle-aged women, and the rate of change accelerates near menopause, especially in blood lipids. Although habitual PA associated with a healthier blood lipid profile and lower body adiposity in middle-aged women in this study, it did not significantly modulate the menopause-related changes in most of the studied metabolic health indicators. However, higher PA may attenuate the increase in SBP and associate with an accelerated increase in WHR. These results indicate that significant increases in PA around menopause may be needed to counteract the menopause-related changes in blood-based biomarkers and body adiposity. Nonetheless, our findings could encourage professionals working with menopausal women to highlight the importance of PA in the early prevention of hypertension and cardiovascular disease. Further longitudinal studies on the role of PA on the metabolic health during the menopausal transition are needed.

## Supplementary information

Supplementary Information (PDF)

## Footnotes

1 The threshold of 0.001 g was determined based on the correspondence with the self-reported wear time in this population (*r* = 0.70). Self-reported wear time was not used in the analysis due to the invalid and missing entries in the diaries.

2 Seven valid days were recorded in 89% (462/528) of the measurements.
