# Supplementary material for: Metabolic health, menopause, and physical activity—a 4-year follow-up study
Source: Int J Obes (Lond). 2021 Nov 20;46(3):544–54. doi: 10.1038/s41366-021-01022-x (PMC8605777; doi:10.1038/s41366-021-01022-x)
Supplement: Supplementary file 1 — Supplementary Information (PDF) [file 41366_2021_1022_MOESM1_ESM.pdf]

Supplementary table 1. Characteristics of the study population in additional physical activity measures and medications

	**Full sample**			**PRE-POST**			**PRE-PRE**			**POST-POST**		
	BL	FU	*Change*[a]	BL	FU	*Change*[a]	BL	FU	*Change*[a]	BL	FU	*Change*[a]
**Self-reported PA [n]**	274	298	*274*	143	149	*143*	53	56	*53*	78	93	*78*
SR-PA [MET-h/day]	5.15 ±4.38	4.91 ±3.93	*-0.23* ±3.72	5.25 ±4.54	4.90 ±3.85	*-0.39 3.71*	5.86 ±4.91	5.81 ±3.90	*-0.10* ±3.26	4.49 ±3.59	4.40 ±4.00	*-0.03* ±*4.05*
**Accelerometer-measured PA [n]**	235	283	*222*	134	141	*126*	43	55	*43*	58	87	*53*
ACC-MVPA [min/day]	67.3 ±29.2	62.8 ±27.2	-3.9 ±23.0	65.7 ±32.5	62.7 ±27.6	-3.1 ±25.3	72.6 ±23.0	68.0 ±25.9	-5.4 ±20.8	67.3 ±25.1	59.8 ±27.2	-4.6 ±19.0
**Medications**[b]												
Use of antihypertensives												
Non-user	86 (238)	78 (233)		90 (129)	79 (117)		83 (44)	80 (45)		82 (65)	76 (71)	
User	14 (38)	22 (65)		10 (15)	21 (32)		17 (9)	20 (11)		18 (14)	24 (22)	
Use of lipid modifying agents												
Non-user	97 (267)	92 (274)		98 (141)	92 (140)		94 (50)	89 (50)		96 (76)	90 (84)	
User	3 (9)	8 (24)		2 (3)	8 (9)		6 (3)	11 (6)		4 (3)	10 (9)	
Use of thyroid therapy												
Non-user	91 (250)	88 (262)		93 (134)	91 (136)		87 (46)	82 (46)		89(70)	86 (80)	
User	9 (26)	12 (36)		7 (10)	9 (13)		13 (7)	18 (10)		11 (9)	14 (13)	

Data are mean ± SD unless otherwise specified. PRE-POST, participants who were pre- or perimenopausal at baseline and postmenopausal at follow-up; PRE-PRE, participants who were pre- or perimenopausal in both measurements; POST-POST, participants who were postmenopausal already at baseline; BL, baseline measurement; FU, follow-up measurement; PA, physical activity; SR-PA, self-report physical activity questionnaire; MET; metabolic equivalent; ACC-MVPA, accelerometer-measured daily moderate-to-vigorous physical activity. [a] For participants with baseline and follow-up measurement, [b] Data are % (n).

Supplementary table 2. Pooled fixed effect estimates for blood-based biomarkers with self-reported physical activity (n=298)

	**Total cholesterol [mmol/l]**		**HDL-C [mmol/l]**		**LDL-C [mmol/l]**		**Glucose [mmol/l]**		**Triglycerides [mmol/l]**	
	B	95% CI	B	95% CI	B	95% CI	B	95% CI	B	95% CI
Intercept (PRE-POST)	5.31***	[5.11, 5.51]	1.61***	[1.52, 1.71]	3.15***	[2.98, 3.33]	5.16***	[5.04, 5.27]	1.18***	[1.05, 1.32]
**Main effects**										
Group										
PRE-POST (ref.)	-		-		-		-		-	
PRE-PRE	0.03	[-0.27, 0.34]	-0.05	[-0.20, 0.10]	0.05	[-0.22, 0.32]	0.05	[-0.12, 0.22]	0.03	[-0.18, 0.24]
POST-POST	0.31*	[0.06, 0.56]	0.20**	[0.07, 0.32]	0.18	[-0.04, 0.40]	0.05	[-0.09, 0.19]	0.03	[-0.14, 0.21]
SR-PA [MET-h/day]	-0.02*	[-0.05, -0,00]	0.02***	[0.01, 0.03]	-0.03*	[-0.05, -0.01]	-0.01	[-0.02, 0.00]	-0.02**	[-0.04, -0.01]
Age at baseline [year]	0.03	[-0.02, 0.09]	-0.00	[-0.03, 0.03]	0.02	[-0.03, 0.08]	-0.00	[-0.03, 0.03]	0.03	[-0.01, 0.07]
Use of hormonal preparations										
Non-user (ref.)	-		-		-		-		-	
Progestogen	-0.14	[-0.32, 0.04]	-0.12**	[-0.20, -0.03]	-0.06	[-0.21, 0.10]	0.06	[-0.05, 0.16]	-0.02	[-0.14, 0.10]
Estrogen	-0.24	[-0.73, 0.25]	0.20	[-0.02, 0.42]	-0.25	[-0.67, 0.18]	0.04	[-0.26, 0.33]	-0.22	[-0.53, 0.10]
Progestogen + Estrogen	-0.17	[-0.42, 0.07]	-0.08	[-0.19, 0.03]	-0.15	[-0.36, 0.06]	-0.18*	[-0.33, -0.04]	-0.02	[-0.18, 0.12]
Time (PRE-POST)	0.62***	[0.43, 0.82]	0.28***	[0.19, 0.36]	0.54***	[0.38, 0.71]	0.15*	[0.03, 0.28]	0.23***	[0.10, 0.35]
**Interactions**										
Time × Group										
Time × PRE-POST (ref.)	-		-		-		-		-	
Time × PRE-PRE	-0.25	[-0.52, 0.01]	-0.08	[-0.19, 0.04]	-0.18	[-0.41, 0.05]	-0.06	[-0.22, 0.11]	-0.14	[-0.31, 0.02]
Time × POST-POST	-0.41***	[-0.64, -0.19]	-0.16**	[-0.25, -0.06]	-0.33***	[-0.52, -0.13]	-0.22**	[-0.36, -0.08]	-0.08	[-0.22, 0.06]
Time × SR-PA	-0.00	[-0.03, 0.02]	-0.01	[-0.02, 0.00]	-0.01	[-0.03, 0.02]	-0.00	[-0.02, 0.01]	-0.00	[-0.01, 0.02]

HDL-C, high-density lipoprotein cholesterol; LDL-C, low-density lipoprotein cholesterol; CI, Confidence interval; PRE-POST, participants who were pre- or perimenopausal at baseline and postmenopausal at follow-up (reference group); PRE-PRE, participants who were pre- or perimenopausal in both measurements; POST-POST, participants who were postmenopausal already at baseline; SR-PA, self-reported physical activity; MET, metabolic equivalent; Time, from baseline to follow-up. * $p \leq 0.05$, ** $p \leq 0.01$, *** $p < 0.001$.

Supplementary table 3. Pooled fixed effect estimates for blood-based lipid biomarkers without participants using lipid modifying agents (n=273)

	**Total cholesterol [mmol/l]**		**HDL-C [mmol/l]**		**LDL-C [mmol/l]**		**Triglycerides [mmol/l]**	
	B	95% CI	B	95% CI	B	95% CI	B	95% CI
Intercept (PRE-POST)	5.46***	[5.12, 5.80]	1.54***	[1.36, 1.73]	3.27***	[2.98, 3.57]	1.22***	[1.01, 1.43]
**Main effects**								
Group								
PRE-POST (ref.)	-		-		-		-	
PRE-PRE	0.05	[-0.26, 0.36]	-0.04	[-0.20, 0.12]	0.06	[-0.21, 0.34]	0.03	[-0.16, 0.23]
POST-POST	0.34*	[0.09, 0.60]	0.19**	[0.06, 0.32]	0.20	[-0.02, 0.43]	-0.01	[-0.17, 0.15]
ACC-MAD [10 mg]	-0.10*	[-0.20, -0,00]	0.06*	[0.01, 0.12]	-0.10*	[-0.19, -0.02]	-0.07*	[-0.13, -0.00]
Age at baseline [year]	0.03	[-0.03, 0.09]	0.00	[-0.03, 0.04]	0.02	[-0.04, 0.07]	0.03	[-0.01, 0.06]
Use of hormonal preparations								
Non-user (ref.)	-		-		-		-	
Progestogen	-0.14	[-0.31, 0.03]	-0.12**	[-0.21, -0.03]	-0.05	[-0.20, 0.10]	-0.00	[-0.11, 0.11]
Estrogen	-0.07	[-0.53, 0.39]	0.17	[-0.07, 0.42]	-0.04	[-0.43, 0.35]	-0.15	[-0.45, 0.14]
Progestogen + Estrogen	-0.27*	[-0.49, -0.05]	-0.09	[-0.21, 0.02]	-0.23*	[-0.42, -0.04]	-0.00	[-0.15, 0.14]
Time (PRE-POST)	0.64***	[0.30, 0.98]	0.38***	[0.20, 0.56]	0.54***	[0.25, 0.84]	0.27*	[0.05, 0.49]
**Interactions**								
Time × Group								
Time × PRE-POST (ref.)	-		-		-		-	
Time × PRE-PRE	-0.18	[-0.42, 0.06]	-0.08	[-0.21, 0.05]	-0.14	[-0.34, 0.07]	-0.17*	[-0.32, -0.01]
Time × POST-POST	-0.37***	[-0.57, -0.17]	-0.15**	[-0.26, -0.04]	-0.28**	[-0.45, -0.11]	-0.09	[-0.22, 0.04]
Time × ACC-MAD	0.01	[-0.10, 0.12]	-0.05	[-0.10, 0.01]	0.01	[-0.09, 0.10]	-0.01	[-0.08, 0.06]

HDL-C, high-density lipoprotein cholesterol; LDL-C, low-density lipoprotein cholesterol; CI, Confidence interval; PRE-POST, participants who were pre- or perimenopausal at baseline and postmenopausal at follow-up (reference group); PRE-PRE, participants who were pre- or perimenopausal in both measurements; POST-POST, participants who were postmenopausal already at baseline; ACC-MAD, accelerometer-measured mean amplitude deviation; mg, milligravity (0.00981 m/s^2); Time, from baseline to follow-up. * $p \leq 0.05$, ** $p \leq 0.01$, *** $p < 0.001$.

Supplementary table 4. Pooled fixed effect estimates for body composition and anthropometrics with self-reported physical activity (n=298)

	**Total fat mass [kg]**		**Android fat mass [kg]**		**Waist circumference [cm]**		**Waist-to-hip ratio ×100**	
	B	95% CI	B	95% CI	B	95% CI	B	95% CI
Intercept (PRE-POST)	25.70***	[24.20, 27.20]	2.31***	[2.14, 2.48]	84.18***	[82.38, 85.97]	83.80***	[82.57, 85.03]
**Main effects**								
Group								
PRE-POST (ref.)	-		-		-		-	
PRE-PRE	0.51	[-2.23, 3.64]	0.04	[-0.26, 0.35]	2.02	[-1.21, 5.24]	1.12	[-0.88, 3.11]
POST-POST	-0.96	[-3.22, 1.30]	-0.08	[-0.33, 0.18]	-1.01	[-3.68, 1.67]	-0.31	[-2.00, 1.38]
SR-PA [MET-h/day]	-0.25***	[-0.36, -0.14]	-0.03***	[-0.04, -0.02]	-0.28***	[-0.43, -0.13]	-0.29***	[-0.44, -0.14]
Age at baseline [year]	0.40	[-0.17, 0.97]	0.04	[-0.02, 0.11]	0.62	[-0.05, 1.28]	0.32	[-0.07, 0.71]
Use of hormonal preparations								
Non-user (ref.)	-		-		-		-	
Progestogen	-0.70	[-1.62, 0.22]	-0.07	[-0.19, 0.04]	-0.36	[-1.53, 0.81]	-0.04	[-1.08, 1.01]
Estrogen	0.23	[-1.80, 2.26]	-0.02	[-0.28, 0.24]	0.19	[-2.49, 2.88]	0.28	[-2.80, 2.25]
Progestogen + Estrogen	-0.67	[-1.84, 0.51]	-0.15*	[-0.29, -0.00]	0.56	[-1.99, 0.88]	0.52	[-0.81, 1.84]
Time (PRE-POST)	2.87***	[2.10, 3.64]	0.39***	[0.29, 0.49]	1.49**	[0.48, 2.50]	0.60	[-0.40, 1.60]
**Interactions**								
Time × Group								
Time × PRE-POST (ref.)	-		-		-		-	
Time × PRE-PRE	-1.09*	[-2.14, -0.04]	-0.12	[-0.26, 0.01]	-0.05	[-1.36, 1.45]	0.70	[-0.71, 2.11]
Time × POST-POST	-1.15*	[-2.05, -0.26]	-0.11	[-0.23, 0.01]	-0.42	[-1.65, 0.82]	0.56	[-0.69, 1.80]
Time × SR-PA	-0.09	[-0.20, 0.02]	-0.01	[-0.02, 0.00]	-0.07	[-0.22, 0.08]	0.15	[-0.00, 0.31]

CI, Confidence interval; PRE-POST, participants who were pre- or perimenopausal at baseline and postmenopausal at follow-up (reference group); PRE-PRE, participants who were pre- or perimenopausal in both measurements; POST-POST, participants who were postmenopausal already at baseline; SR-PA, self-reported physical activity; MET, metabolic equivalent; Time, from baseline to follow-up. * $p \leq 0.05$, ** $p \leq 0.01$, *** $p < 0.001$.

Supplementary table 5. Pooled fixed effect estimates for blood pressure with self-reported physical activity (n=298)

	**Systolic blood pressure [mmHg]**		**Diastolic blood pressure [mmHg]**	
	B	95% CI	B	95% CI
Intercept (PRE-POST)	131.97***	[128.36, 135.57]	84.59***	[82.66, 86.51]
**Main effects**				
Group				
PRE-POST (ref.)	-		-	
PRE-PRE	1.50	[-4.21, 7.22]	1.16	[-1.99, 4.31]
POST-POST	-1.98	[-6.81, 2.85]	-0.11	[-2.78, 2.56]
SR-PA [MET-h/day]	-0.03	[-0.43, 0.38]	-0.14	[-0.34, 0.06]
Age at baseline [year]	1.15*	[0.03, 2.27]	0.42	[-0.21, 1.06]
Use of hormonal preparations				
Non-user (ref.)	-		-	
Progestogen	0.25	[-2.90, 3.41]	-0.29	[-1.91, 1.32]
Estrogen	-0.10	[-8.04, 7.85]	-1.68	[-5.57, 2.22]
Progestogen + Estrogen	-5.50**	[-9.59, -1.42]	-4.32***	[-6.35, -2.29]
Time (PRE-POST)	4.57**	[1.46, 7.69]	-0.10	[-1.62, 1.42]
**Interactions**				
Time × Group				
Time × PRE-POST (ref.)	-		-	
Time × PRE-PRE	0.74	[-3.58, 5.06]	-0.61	[-2.67, 1.46]
Time × POST-POST	-0.34	[-4.14, 3.46]	-0.78	[-2.63, 1.06]
Time × SR-PA	-0.41	[-0.85, 0.02]	-0.17	[-0.39, 0.05]

CI, Confidence interval; PRE-POST, participants who were pre- or perimenopausal at baseline and postmenopausal at follow-up (reference group); PRE-PRE, participants who were pre- or perimenopausal in both measurements; POST-POST, participants who were postmenopausal already at baseline; SR-PA, self-reported physical activity; MET, metabolic equivalent; Time, from baseline to follow-up. * $p \leq 0.05$, ** $p \leq 0.01$, *** $p < 0.001$.

Supplementary table 6. Pooled fixed effect estimates for blood pressure without participants using antihypertensives (n=230)

	**Systolic blood pressure [mmHg]**		**Diastolic blood pressure [mmHg]**	
	B	95% CI	B	95% CI
Intercept (PRE-POST)	126.67***	[120.42, 132.90]	82.01***	[78.85, 85.17]
**Main effects**				
Group				
PRE-POST (ref.)	-		-	
PRE-PRE	-0.30	[-6.48, 5.88]	0.68	[-2.71, 4.08]
POST-POST	-1.07	[-6.27, 4.13]	0.30	[-2.54, 3.15]
ACC-MAD [10 mg]	0.66	[-1.15, 2.46]	-0.07	[-0.95, 0.82]
Age at baseline [year]	0.69	[-0.55, 1.92]	0.16	[-0.53, 0.86]
Use of hormonal preparations				
Non-user (ref.)	-		-	
Progestogen	0.71	[-2.60, 4.03]	-0.10	[-1.75, 1.54]
Estrogen	1.51	[-6.12, 9.15]	-1.09	[-4.73, 2.55]
Progestogen + Estrogen	-4.28	[-8.72, 0.16]	-4.26***	[-6.43, -2.09]
Time (PRE-POST)	11.16**	[4.63, 17.70]	1.37	[-1.64, 4.39]
**Interactions**				
Time × Group				
Time × PRE-POST (ref.)	-		-	
Time × PRE-PRE	0.98	[-3.58, 5.55]	-1.03	[-3.13, 1.08]
Time × POST-POST	-1.28	[-5.19, 2.63]	-1.71	[-3.52, 0.10]
Time × ACC-MAD	-2.52*	[-4.64, -0.38]	-0.48	[-1.46, 0.51]

CI, Confidence interval; PRE-POST, participants who were pre- or perimenopausal at baseline and postmenopausal at follow-up (reference group); PRE-PRE, participants who were pre- or perimenopausal in both measurements; POST-POST, participants who were postmenopausal already at baseline; ACC-MAD, accelerometer-measured mean amplitude deviation; mg, milligravity (0.00981 m/s^2); Time, from baseline to follow-up. * $p \leq 0.05$, ** $p \leq 0.01$, *** $p < 0.001$.

Supplementary table 7. Pooled fixed effect estimates for number of metabolic syndrome risk factors with self-reported physical activity (n=298)

	**Number of metabolic syndrome risk factors**	
	exp(B)	95% CI
Intercept (PRE-POST)	1.41**	[1.11, 1.80]
**Main effects**		
Group		
PRE-POST (ref.)	-	
PRE-PRE	1.33	[0.95, 1.85]
POST-POST	0.92	[0.69, 1.22]
SR-PA [MET-h/day]	0.94*	[0.91, 0.97]
Age at baseline [year]	1.07*	[1.01, 1.14]
Use of hormonal preparations		
Non-user (ref.)	-	
Progestogen	0.99	[0.79, 1.23]
Estrogen	0.68	[0.34, 1.39]
Progestogen + Estrogen	0.71	[0.50, 1.02]
Time (PRE-POST)	1.20	[0.92, 1.58]
**Interactions**		
Time × Group		
Time × PRE-POST (ref.)	-	
Time × PRE-PRE	0.79	[0.54, 1.16]
Time × POST-POST	0.94	[0.68, 1.30]
Time × SR-PA	0.99	[0.95, 1.03]

CI, Confidence interval; PRE-POST, participants who were pre- or perimenopausal at baseline and postmenopausal at follow-up (reference group); PRE-PRE, participants who were pre- or perimenopausal in both measurements; POST-POST, participants who were postmenopausal already at baseline; SR-PA, self-reported physical activity; MET, metabolic equivalent; Time, from baseline to follow-up; * $p \leq 0.05$, ** $p \leq 0.01$, *** $p < 0.001$.

Supplementary table 8. Pooled fixed effect estimates for number of metabolic syndrome risk factors without participants using lipid modifying agents or antihypertensives (n=220)

	**Number of metabolic syndrome risk factors**	
	exp(B)	95% CI
Intercept (PRE-POST)	1.27	[0.73, 2.20]
**Main effects**		
Group		
PRE-POST (ref.)	-	
PRE-PRE	1.29	[0.84, 1.98]
POST-POST	0.95	[0.65, 1.37]
ACC-MAD [10 mg]	0.88	[0.74, 1.04]
Age at baseline [year]	1.04	[0.96, 1.12]
Use of hormonal preparations		
Non-user (ref.)	-	
Progestogen	0.98	[0.74, 1.30]
Estrogen	0.91	[0.38, 2.15]
Progestogen + Estrogen	0.69	[0.44, 1.08]
Time (PRE-POST)	1.57	[0.80, 3.11]
**Interactions**		
Time × Group		
Time × PRE-POST (ref.)	-	
Time × PRE-PRE	0.77	[0.48, 1.26]
Time × POST-POST	0.96	[0.63, 1.47]
Time × ACC-MAD	0.88	[0.69, 1.11]

CI, Confidence interval; PRE-POST, participants who were pre- or perimenopausal at baseline and postmenopausal at follow-up (reference group); PRE-PRE, participants who were pre- or perimenopausal in both measurements; POST-POST, participants who were postmenopausal already at baseline; ACC-MAD, accelerometer-measured mean amplitude deviation; mg, milligravity (0.00981 m/s^2); Time, from baseline to follow-up. * $p \leq 0.05$, ** $p \leq 0.01$, *** $p < 0.001$.
